# Factors that Influence Adherence to Antiretroviral Treatment in an Urban Population, Jakarta, Indonesia

**DOI:** 10.1371/journal.pone.0107543

**Published:** 2014-09-17

**Authors:** Emma Rosamond Nony Weaver, Masdalina Pane, Toni Wandra, Cicilia Windiyaningsih, Herlina, Gina Samaan

**Affiliations:** 1 Sulianti Saroso Infectious Diseases Hospital, Ministry of Health Republic of Indonesia, North Jakarta, Jakarta Province, Indonesia; 2 National Centre for Epidemiology and Population Health, Australian National University, Canberra, Australian Capital Territory, Australia; 3 Independent Researcher, Jakarta, Indonesia; Fundacion Huesped, Argentina

## Abstract

**Introduction:**

Although the number of people receiving antiretroviral therapy (ART) in Indonesia has increased in recent years, little is known about the specific characteristics affecting adherence in this population. Indonesia is different from most of its neighbors given that it is a geographically and culturally diverse country, with a large Muslim population. We aimed to identify the current rate of adherence and explore factors that influence ART adherence.

**Methods:**

Data were collected from ART-prescribed outpatients on an HIV registry at a North Jakarta hospital in 2012. Socio-demographic and behavioral characteristics were explored as factors associated with adherence using logistics regression analyses. Chi squared test was used to compare the difference between proportions. Reasons for missing medication were analyzed descriptively.

**Results:**

Two hundred and sixty-one patients participated, of whom 77% reported ART adherence in the last 3 months. The level of social support experienced was independently associated with adherence where some social support (p = 0.018) and good social support (p = 0.039) improved adherence compared to poor social support. Frequently cited reasons for not taking ART medication included forgetting to take medication (67%), busy with something else (63%) and asleep at medication time (60%).

**Discussion:**

This study identified that an increase in the level of social support experienced by ART-prescribed patients was positively associated with adherence. Social support may minimize the impact of stigma among ART prescribed patients. Based on these findings, if social support is not available, alternative support through community-based organizations is recommended to maximize treatment success.

## Introduction

Antiretroviral therapy (ART) has dramatically reduced rates of morbidity and mortality among HIV infected persons worldwide, transforming the disease from a fatal illness to a manageable chronic condition [1], [2]. While this is a huge achievement, ART treatment success– defined as sustained virologic suppression and immunologic recovery, depends fundamentally on patient adherence to treatment [1], [3]. Suboptimal adherence can lead to inadequate viral load suppression, the emergence of resistance, treatment failure in patients and the potential transmission of drug resistant virus strains back into the community [2]. WHO guidelines recommend at least 95% adherence to ART prescribed medication [4].

Although the importance of ART adherence is widely recognized, its practice remains a challenge. Very high levels of adherence are particularly recommended in developing countries where less robust lines of treatment, such as non-nucleoside reverse transcriptase inhibitors (NNRTI), are commonly used [5]. However socio-demographic, cultural, economic and health care systems related factors are proven barriers to successful adherence. Indonesian studies have shown that stigma and discrimination can further impede adherence by forcing HIV-infected patients to keep their status a secret for fear of being outcaste by their community and bringing shame on the family [6], [7].

In a recent global systematic review of factors causing ART interruption the most frequently reported reasons were related to drug toxicity and side effects, with developing countries additionally referencing the cost of ART treatment and frequent pharmacy stock-outs as further barriers [8]. Another recent systematic review of studies across Asia showed that poor adherence was largely due to the financial burden of ART treatment, with travel and diagnostic costs further impeding access [9]. A Nepalese study found that non-disclosure of HIV status, alcohol use and being female as major prohibiting factors [10] while an Indian study showed that a lack of social support and in particular a lack of reminders from family to take medication were negatively associated with adherence [11]. To date no studies have been published on risk factors for ART interruption in Indonesia.

Although the number of people receiving ART in Indonesia has increased in recent years, from 2,381 in 2006 to 24,410 in 2011 [12], [13], little is known about the specific characteristics affecting levels of adherence in this population. Indonesia is different from most of its neighbors given that it is a geographically and culturally diverse country, with a large Muslim population of which many fast during Ramadan every year. The impact of the religious and cultural diversity on ART adherence is not well established, but warrants investigation.

**Figure 1:**
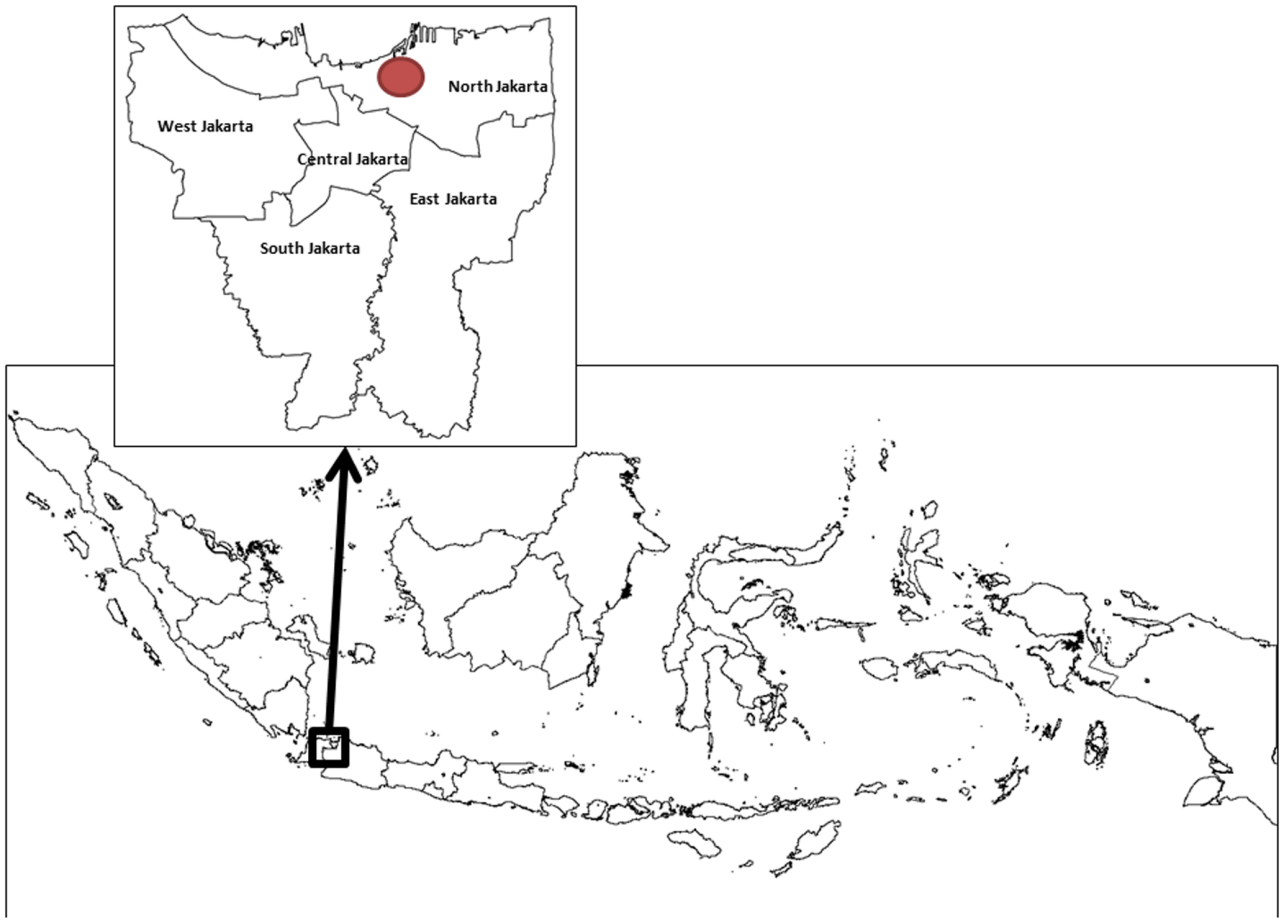
Location of Sulianti Saroso Infectious Disease Hospital in North Jakarta, Indonesia.

Sulianti Saroso Infectious Disease Hospital is one of the national referral hospitals for HIV-AIDS, located in North Jakarta (Figure 1). The hospital currently serves more than 1,300 ART prescribed patients. The hospital also provides stocks of ART and training to primary health clinics that administer ART to patients on their registries. At this hospital, nurses have reported poor monthly re-attendance by ART prescribed patients to collect their ART medication from the pharmacy service. This is used by the hospital as a proxy to gauge adherence rates as outpatients are given only one-month supply of medication at each visit. This, combined with frequent self-reporting of poor adherence by patients on ART for more than one year, was the rationale for undertaking this study.

This study draws on this hospital-based sample of ART prescribed patients at Sulianti Saroso Infectious Disease Hospital in North Jakarta. We aimed to identify the current rate of adherence and explore factors that influence adherence in this setting.

## Methods

### Ethics

Ethics approval for this study was obtained from University of Indonesia with the approval number 486/PT02.FK/ETIK/2012. Written consent was also obtained from respondents at time of interview.

### Study design and setting

A cross-sectional study was conducted between August and November 2012 and included a quantitative survey of ART prescribed patients from Sulianti Saroso Infectious Disease Hospital. This hospital was selected because of its long experience in HIV case management, the presence of an HIV specialist team as well as an in-house research unit, and because it is one of the most prominent ART administering sites in Jakarta.

### Data collection

Respondents were taken from the hospital HIV outpatient registry and considered eligible if ≥ 18 years old and on ART for at least 6 months. Nurses explained the objectives of the study to patients collecting their ART medication from the hospital and asked for their consent to participate. Of respondents who agreed to participate, they were then asked to complete a questionnaire in a private room with a nurse present. This questionnaire assessed socio-demographic characteristics, access to health facilities, knowledge of HIV and ART, and adherence in the last four days and last three months. Patients were considered adherent based on two measures- if they self-reported taking ≥ 95% of ART medication in a 4-day (missing ≥ 1 dose of ART medication within this time period was determined as non-adherent) and a 3-month (missing ≥ 3 doses of ART medication within this time period was determined as non-adherent) recall period immediately prior to the interview date [10], [14].

Respondents were asked about social support and how they perceive themselves (personal stigma) and how they think others perceive them (external stigma) due to their HIV status. The Interpersonal Support Evaluation List (ISEL) - a 40-item Likert scale with responses from 0 “definitely false” to 3 “definitely true”- was adapted to exclude one question that was deemed culturally inappropriate and used to determine the level of social support experienced by respondents [15]. Negative items were reverse coded and scores ranged from 0 to 117 with higher scores suggesting greater perceived social support. The Perceived Social Stigma (PSS) tool - also a 40-item Likert scale with responses from 1 “strongly disagree” to 4 “strongly agree”– was adapted and all 40 questions were used to determine the level of personal and external stigma [15]. Total scores ranged from 0 to 160, with higher scores indicating more stigma.

### Analysis

To assess factors associated with adherence, the dependent variable was ‘adherence to ART’ using a 3-month recall period and independent variables included socio-demographic and cultural variables, access to health facilities, knowledge of ART, personal and external stigma and social support. See Study Dataset S1 for the dataset used in this study.

The ISEL tool does not prescribe cut offs for levels of social support experienced and therefore for our analyses scores were categorized into three groups. The 3 groups for social support were “poor” =  60 or less, “some” =  61–70 and “good” =  71 or more. For the PSS scale, questions were divided into two groups for analysis- those that focused on personal stigma and those that focused on external stigma. For the personal stigma assessment scores ranged from 0 to 95 (with scores between 0 and 46 indicating less stigma) and for the external stigma assessment scores ranged from 0 to 153 (with scores between 0 and 34 indicating less stigma) [15].

Chi squared test was used to compare the difference between proportions. Logistic regression analysis was performed to determine variables associated with adherence. The dependent variable was coded as binary (0 = adherent and 1 = non-adherent). Univariable analyses were initially conducted and all variables with p-value ≤0.1 were entered into a multiple logistic regression model. Backward stepwise selection was used to determine variables independently associated with adherence at p<0.05 level. All analyses were conducted using Stata V.11 [16].

## Results

### Quantitative results

In total 276 ART prescribed patients were approached and 261 agreed to participate (95%, Table 1); 74% were male and the mean age was 33.4 years (range 20 to 61). The majority (60%) of respondents had completed secondary school, 54% were married and 41% were unemployed. Of all respondents, 29% reported ever injecting drugs. The majority (73%) were Muslim, of whom 51% stated they fast during Ramadan every year, 45% travel more than 10 km to collect ART medication and the median time since HIV diagnosis was 50.1 months (IQR 29.3–65.8). The median duration on ART was 45.7 months (IQR 25.7–60.7). The median duration since last CD4 count test was 10.2 months (IQR 5.2–19.8) with 44% of respondents reporting that their last CD4 count test was over 1 year ago. Of patients, 13% reported ever having an opportunistic infection since their HIV diagnosis.

**Table 1:** Socio-demographic characteristics of ART prescribed patients at Sulianti Saroso Infectious Disease Hospital, Jakarta, N = 261.

Variable	N	%
**Sex**		
Male	193	73.9
Female	68	26.1
**Age**		
30 and under	91	34.9
31 to 40	141	54.0
41 and over	29	11.1
**Marital status**		
Not married	82	31.4
Married	142	54.4
Divorced/widowed	27	14.2
**Children**		
Yes	145	55.6
No	116	44.4
**Education**		
Primary	40	15.3
Secondary	157	60.2
Tertiary	64	24.5
**Hours worked**		
Full time	109	41.8
Part time	44	16.9
Not working	108	41.4
**History of injecting drug use**		
Yes	76	29.1
**Religion**		
Muslim	191	73.2
Catholic	47	18.0
Other	23	8.8
**Ramadan fasting (N = 191 Muslims)**		
Yes	98	51.3
No	93	48.7
**HIV diagnosis (months)** Median (IQR)	50.1 (29.3–65.8)	
**ART initiated (months)** median (IQR)	45.7 (25.7–60.7)	
**Last CD4 count (months)** median (IQR)	10.3 (5.2–19.8)	
**Co-infection**	35	13.4
Yes		
**Distance to medication**		
10 km or less	143	54.8
Over 10 km	118	45.2
**Self-reported adherence**		
Last 4 days	233	89.3
Last 3 months	200	76.6
**Stigma**		
Experienced personal stigma	218	83.5
Experienced external stigma	202	77.4
**Social support**		
Poor (60 or less)	26	10.0
Some (61–70)	98	37.6
Good (71 or more)	137	52.5

Most (89%) respondents reported ART adherence in the last 4 days with fewer reporting adherence in the last 3 months (77%; p<0.001). The majority also reported experiencing stigma because of their HIV status, with 84% reporting personal stigma and 77% reporting external stigma. The majority (52%) reported good social support while 38% and 10% reported some and poor social support respectively.

### Factors associated with adherence to ART

Four variables had *P* values ≤0.1 on univariable analyses (Table 2): marital status, education, fasting and social support. Age, sex, number of children, number of people in household, hours worked, injecting drug use, religion, total monthly household expenditure and distance to medication were not associated with adherence (data not shown).

**Table 2:** Relationship between patient characteristics and three month drug adherence, N = 261.

	Adherence (%)		Univariable		Mulitvariate	
Variable	Not adherent	Adherent	OR (95% CI)^a^	P-value	OR (95% CI)^a^	P-value
**Gender**						
Male	44 (22.8)	149 (77.2)				
Female	17 (25)	51 (75)	1.129 (0.59–2.15)	0.712		
**Age**						
30 and under	22 (24.2)	69 (75.8)				
31 to 40	32 (22.7)	109 (77.3)	0.921 (0.49–1.71)	0.794		
41 and over	7 (24.1)	22 (75.9)	0.998 (0.38–2.65)	0.997		
**Marital status**						
Not married	18 (21.9)	64 (78.1)				
Married	32 (22.5)	110 (77.5)	3.048 (0.07–0.91)	0.071		
Divorced/widowed	11 (29.7)	26 (70.3)	0.936 (0.91–0.31)	0.907		
**Children**						
No	35 (24.1)	110 (75.9)				
Yes	26 (22.4)	90 (77.6)	1.482 (0.63–3.55)			
**Education**						
Primary	13 (32.5)	27 (67.5)				
Secondary	29 (18.5)	128 (81.5)	0.471 (0.22–1.02)	0.039		
Tertiary	19 (29.7)	45 (70.3)	0.877 (0.37–2.10)	0.545		
**Work**						
Not working	31 (20.3)	122 (79.7)				
Working	30 (27.8)	78 (72.2)	0.661 (0.37–1.18)	0.159		
**Religion**						
Buddhist	3 (27.3)	8 (72.7)				
Catholic	8 (13.6)	51 (86.4)	0.418 (0.09–1.92)	0.262		
Muslim	50 (26.2)	141 (73.8)	0.946 (0.24–3.70)	0.936		
**Fasting**						
No	32 (19.6)	131 (80.4)				
Yes	29 (29.6)	69 (70.4)	1.721 (0.96–3.08)	0.067		
**Distance to medication**						
10 km or less	35 (24.5)	108 (75.4)				
Over 10 km	26 (22.0)	92 (78.0)	0.872 (0.49–1.56)	0.643		
**Total monthly household expenditure** b						
1 000 000 or less	11 (19.3)	46 (80.7)				
Between 1 and 2 million	23 (28.6)	57 (71.4)	1.687 (0.75–3.82)	0.209		
More than 2 million	22 (27.2)	59 (71.8)	1.559 (0.69–3.54)	0.288		
**Level of social support received from others**						
Poor (60 or less)	11 (42.3)	15 (57.7)				
Some (61–70)	19 (19.4)	79 (80.6)	0.328 (0.130–0.827)	0.018	0.328 (0.130–0.827)	0.018
Good (71 or more)	31 (22.6)	106 (77.4)	0.399 (0.166–0.957)	0.039	0.399 (0.166–0.957)	0.039

a OR odds ratio; CI confidence interval.

b Data missing (total monthly expenditure n = 218).

Multivariable analysis (Table 2) showed that the level of social support experienced by respondents affected adherence; greater levels of social support significantly improved adherence. Using poor social support as the reference group, some social support (p = 0.018) and good social support (p = 0.039) both significantly improved adherence levels.

Of the 61 respondents that reported non-adherence in the last 3 months, 57 identified one or more reasons for non-adherence (Table 3). Frequently cited reasons include forgetting to take medication (67%), busy with something else (63%), asleep at medication time (60%) and ran out of medication (44%). Other common barriers included medication only being available far from house (37%), not wanting others to know they were taking HIV medication (35%) and too many pills to swallow (33%).

**Table 3:** Reasons provided by non-adherent respondents for missing medication, N = 57.

Variable*	N	%
Forgot	38	66.7
Busy with something else	36	63.2
Asleep at time of taking medication	34	59.6
Run out of medication	25	43.9
Far from house	21	36.8
Not wanting others to know taking HIV medication	20	35.1
Too many pills to swallow	19	33.3
Feeling sick or unwell	18	31.6
Feeling depressed or hopeless	18	31.6
Have trouble swallowing medication	14	24.6
Want to avoid the side effects	13	22.8
Already feel healthy	12	21.1
There is a change in routine	11	19.3
Felt the drug was toxic	10	17.5

*Multiple answers allowed.

## Discussion

This study described the self-reported adherence rate in the 3 months prior to the study among ART prescribed patients attending a large infectious disease referral hospital in North Jakarta in 2012. The rate of adherence in our sample (77%) is comparable to other Asian studies using a self-reporting measure for adherence [11], [17], [18]. This rate is also higher than adherence rates reported in some North-American based studies [19]. Possible reasons for these higher rates of adherence are that we relied on self-reporting which can overestimate adherence [20] and that we recruited patients who were actively engaging with a large referral hospital in a metropolitan area.

Our study found that greater social support, as assessed by the Interpersonal Support Evaluation List scale, was significantly associated with adherence, which is consistent with other studies [11], [21]–[23]. This suggests that family and friends play an important role in supporting ART prescribed patients, potentially by reminding them to take their medication, assisting with the collection of ongoing ART medication, increasing their sense of connectedness to others, reducing isolation and providing an incentive outside of themselves to adhere to treatment. Furthermore, social support may minimize the impact of stigma among ART prescribed patients. Although many respondents in our study reported experiencing personal or external stigma, this did not impact their ART adherence.

Based on these findings, if social support is not available, alternative support through community-based organizations should be provided where possible to improve the social networks of ART prescribed patients and maximize treatment success. At Sulianti Saroso Infectious Disease Hospital, there are psychosocial counsellors and HIV infected community groups that provide social to HIV patients. This structure can be used to address social support issues once non-adherent ART prescribed patients are identified. Qualitative studies of the relationship between ART adherence, social support and stigma are recommended in the future.

Unlike previous studies that found low educational attainment to be a predictor for non-adherence, educational attainment was not independently associated with ART adherence in our study. This is a surprising finding as higher levels of education and literacy likely facilitate better communication between the patient and the health worker, increase retention of information provided by the health worker and therefore ensure a greater understanding of how to take ART medication by the patient. Literacy is also empowering, and a lack thereof may result in a reluctance from patients to ask others for help [24]. Further research is needed to explore the relationship between education levels and drug adherence in this hospital setting.

The key reasons for not taking ART medication given by patients were forgetfulness, being busy with something else, sleeping when meant to be taking medication and running out of medication. While behavioral reasons are difficult to address programmatically, increasing awareness of the importance of taking medication as prescribed is essential and must be emphasized in pre-treatment counseling and all subsequent engagements with health services providing ART medication. Patient recall to the clinic to collect new batches of drugs should be actively encouraged.

Despite sickness being a recognized exemption from fasting in Islam, over half of ART prescribed patients in our sample who identified as being Muslim stated they fast every year during Ramadan. Fasting did not have a significant effect on adherence rates which is consistent with other literature from Sub-Saharan Africa [25]. This study showed that patients altered their typical daily ART consumption behaviors by advancing morning and delaying evening doses during Ramadan [25]. Another study looking at the impact of fasting on patients with chronic asthma also found that fasting did not affect adherence to treatment but rather affected the times at which medication was used by patients [26]. Patients in our study may have rearranged their medication consumption times. Where appropriate, counseling for ART treatment needs to include discussion on fasting. If patients plan to fast during Ramadan this needs to be carefully monitored to ensure optimal adherence is maintained. The impact of rescheduling drug therapy on adherence has also not been explored and is recommended in future studies.

An additional finding from this study was that many ART prescribed patients do not receive CD4 count tests annually (44%), although this is recommended in WHO guidelines [27]. Future ART programs need to scale up annual CD4 count testing in their ART prescribed patients to monitor their treatment, and if this is too costly, alternative measures for monitoring viral load need to be utilized.

The main strength to our study is that it is the first to examine adherence to ART in Indonesia. Our study also had a high response rate- fifteen patients (5%) who were asked to participate refused to do so. However the study does have some limitations. Due to financial constraints, adherence was based on self-reporting as opposed to more objective tools such as electronic pill caps or pill counts. While there are concerns that self-reporting reflects only short-term or average adherence and may often overestimate adherence [28], previous studies have determined this to be an appropriate, robust indicator of adherence.

Another limitation to this study is that only patients who attended the hospital were included. Those who did not attend or had others collecting their medication on their behalf were not interviewed and may differ in terms of their adherence and the severity of their disease. This may influence the generalizability of the results. Also given that the focus of this study was to explore adherence, our selection criteria included treatment with ART for a minimum of 6 months, and therefore the proportion of patients with early abandonment was not explored in the study. This study is also a cross-sectional analysis, measuring adherence at a single time point. However adherence is a process that inevitably varies over time, meaning that multiple interviews with patients may have provided greater insight into adherence behavior. Longitudinal studies engaging with a variety of HIV patients in combination with a variety of tools for measuring adherence including grading patient adherence or exploring psychological factors such as depression are recommended in the future, to understand adherence more comprehensively.

Lastly, this study sample is based on patients actively engaging with a hospital in a metropolitan area in the wealthiest province of Indonesia and therefore these findings may not be generalizable to other parts of the country, and in particular to more remote and disadvantaged locations. Further studies are needed in other areas of Indonesia to gain a more accurate understanding of the levels of adherence and how they vary based on geographical, cultural, religious and ethnic differences.

### Conclusion

This study has identified that an increase in the level of social support experienced by ART prescribed patients was positively associated with adherence. Forgetting to take medication, busy during time of taking medication and running out of medication were common reasons for not adhering to treatment. Given that social factors and behavioral patterns influence adherence, interventions need to target these issues in order to maximize treatment success. The focus needs to be on providing opportunities for social networking among ART prescribed patients when needed, eliminating barriers to accessing treatment, monitoring the progress of therapy as well as CD4 count regularly, increasing patient's awareness of ART treatment and improving medical record keeping of patients. Policy makers must work towards policies that encourage patients to achieve optimal adherence levels. Further studies are also recommended in other settings of Indonesia given that geographical, social, cultural, religious and health service access factors differ significantly across the country. Qualitative studies reviewing the relationship between ART adherence, social support and stigma are also recommended.

## Supporting Information

Study Dataset S1 **Dataset used in this study.**
(XLSX)
